# Supplementary material for: Predicting the spatiotemporal diversity of seizure propagation and termination in human focal epilepsy
Source: Nat Commun. 2018 Mar 14;9:1088. doi: 10.1038/s41467-018-02973-y (PMC5852068; doi:10.1038/s41467-018-02973-y)
Supplement: Supplementary file 1 — Supplementary Information [file 41467_2018_2973_MOESM1_ESM.pdf]

Predicting the spatiotemporal diversity of seizure propagation and termination in human focal epilepsy
Supplementary Information

Proix et al.

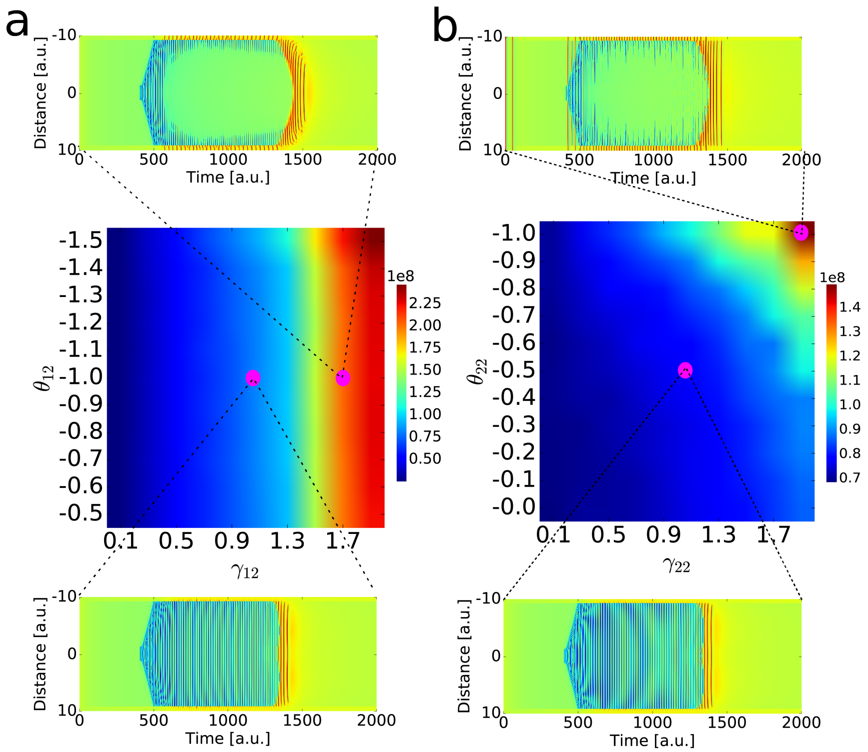

Supplementary Figure 1. Parameter space exploration for $\theta_{12}, \gamma_{12}, \theta_{22}, \gamma_{22}$. Exploration of the other coupling parameters are shown in Figs. 3 and 6 of the main text. (a) Middle plot: parameter space exploration for parameters θ_{12} and γ_{12}. The plot represents the amount of time the second system is above a given threshold (variable $q_1 > 0.4$) over the full simulated field. Blue values correspond to seizures as discussed in the main text (bottom time series with parameters $\theta_{12} = -1.0, \gamma_{12} = 1.1$). For red values, the second population stops oscillating and stays in a high activity state for a significant part of the seizure (upper time series with parameters $\theta_{12} = -1.0, \gamma_{12} = 1.7$). Indeed, for large coupling values ($\gamma_{12} > 1.4$), the unstable fixed point given by the crossing of the green and red nullclines in Fig. 4b of the main text switches to the right branch of the red nullcline during the seizure, and becomes stable through a supercritical Hopf bifurcation. (b) Same as in (a), but for parameters θ_{22} and γ_{22}. Blue values correspond to seizures as discussed in the main text (bottom time series with parameters $\theta_{22} = -0.5, \gamma_{22} = 1.1$). High values of γ_{22} combined with low values of θ_{22} make the second population switch to a high activity state during the seizure (red values, upper time series with parameters $\theta_{22} = -1.9, \gamma_{22} = 1.0$).

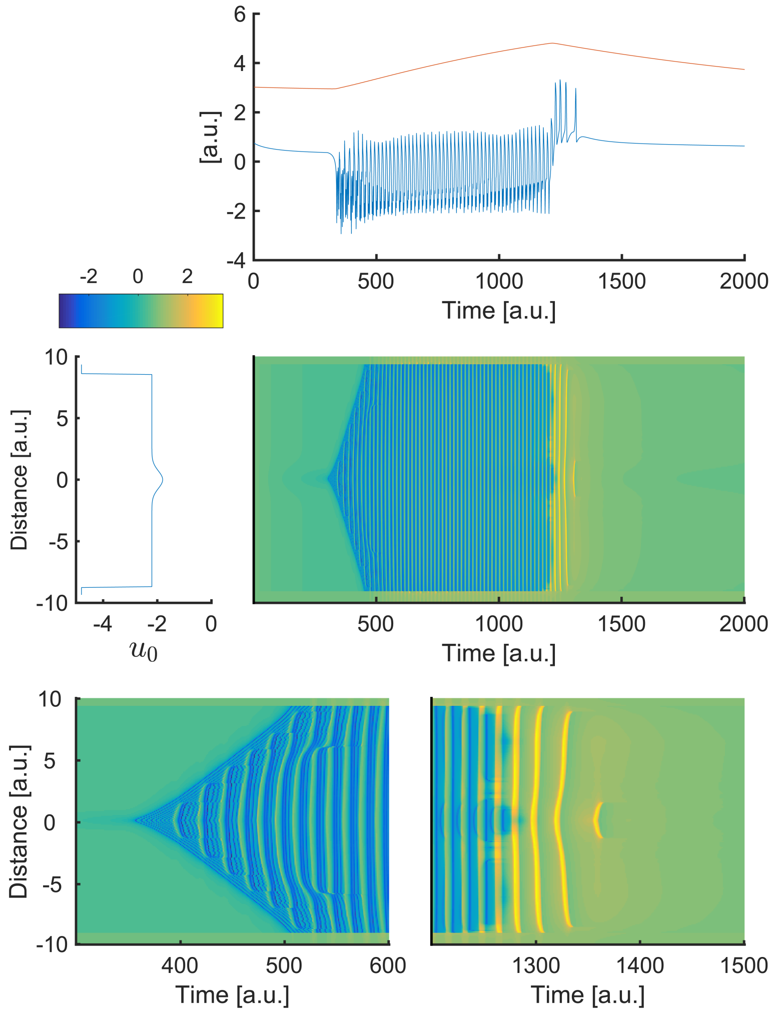

Supplementary Figure 2. The Epileptor field model can spontaneously transition in and out of seizures. The transitions occur without stimulation, but by changes in the spatial values of the excitability parameter u_0. Note that the location of the source of the SWDs stays stationary during the seizure. A single Epileptor field is simulated (middle row). Spatial values for excitability parameter and simulated spatiotemporal activity are shown on the left and right middle row respectively. The seizure starts in the central location of the Epileptor field model where the excitability is higher, and slowly propagates throughout the field. Time series at the zero location (where the seizure first starts) are shown in the top row for the field activity ($-u_1 + q_1$, in blue) and the slow permittivity variable (v, in red). The lower row shows a zoomed-in view of the spatiotemporal activity at seizure onset (left) and offset (right). The seizure ends synchronously in the field, with a few spike-and-wave events propagating after seizure offset. Red dots and the dashed line mark the source location of the propagating SWDs at seizure onset and offset.

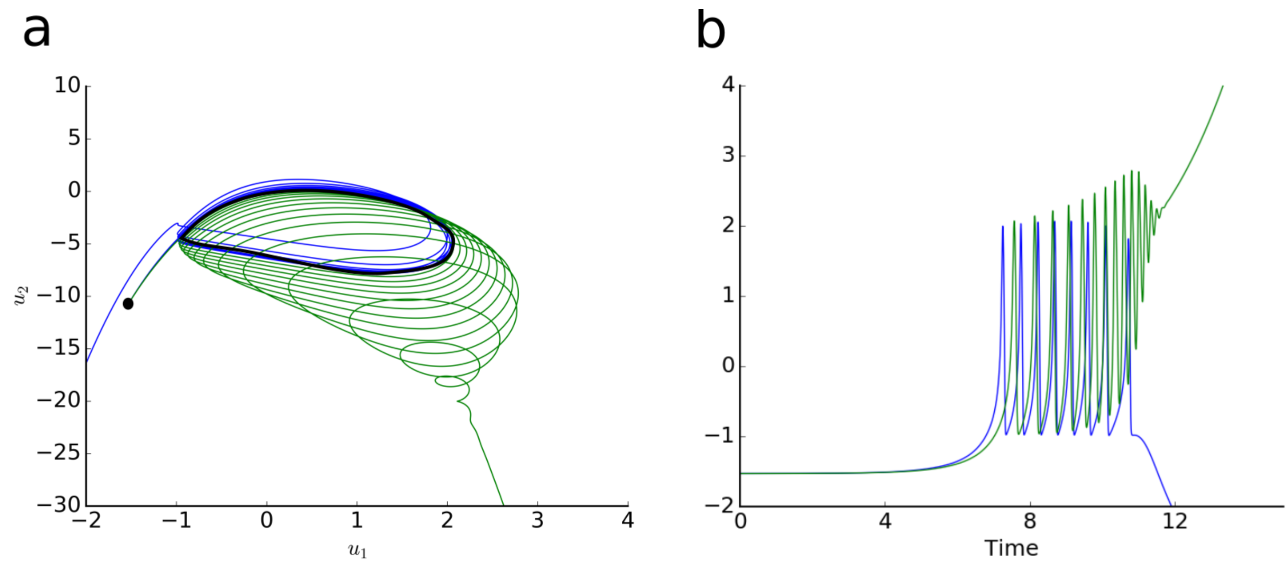

Supplementary Figure 3. Shooting method to compute propagation speed of the fast population. (a) Phase space for the fast population. Black dot: interictal stable fixed point. Black closed curve: stable limit cycle. Simulated trajectories are shown for $c_1 < c_1^*$ (blue) and $c_1 > c_1^*$ (green), with c_1^* the speed of the front. (b) Corresponding time series for $c_1 < c_1^*$ (blue) and $c_1 > c_1^*$ (green).

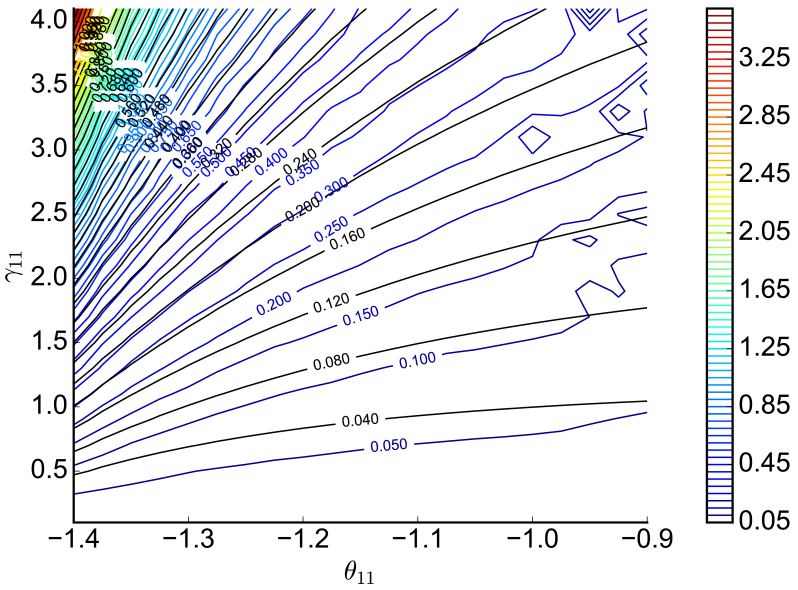

Supplementary Figure 4. Comparison of the speed of propagation for shooting method and simulation of the full model. Shooting method: black lines. Simulation of the full model: colored lines.

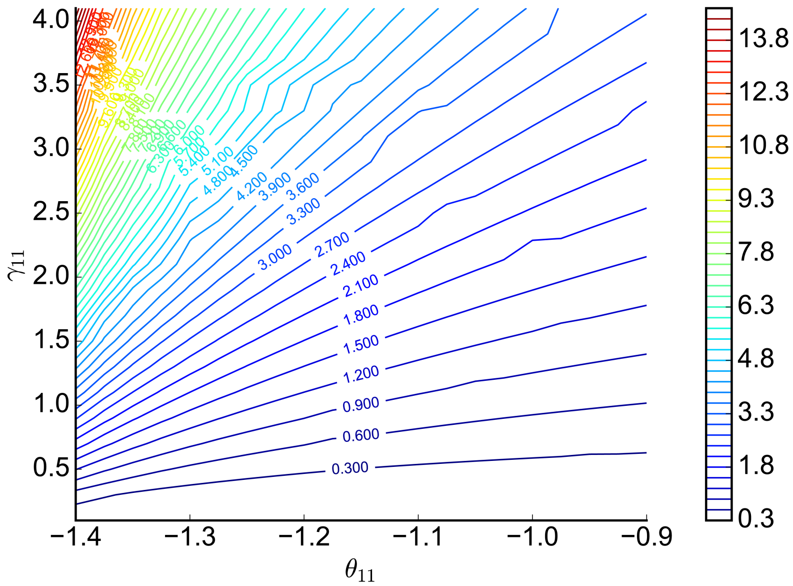

Supplementary Figure 5. Shooting method for the fast population in the reduced model.

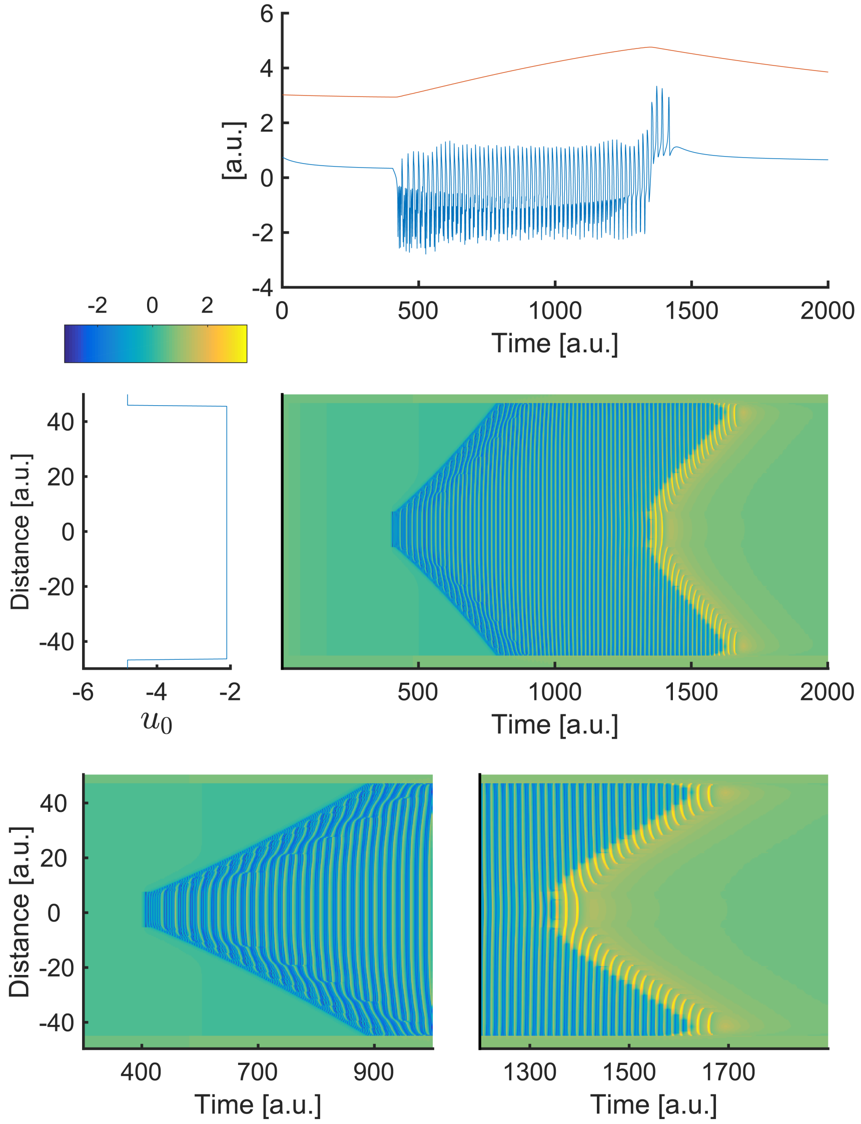

Supplementary Figure 6. Seizures do not end synchronously across the field if the delays of recruitment are too big compared to the length of the seizure. Large delays were here obtained by increasing the spatial domain. A single Epileptor field model is simulated (middle row). Spatial values for excitability parameter and simulated spatiotemporal activity are shown on the left and right middle row respectively. The seizure starts in the central location of the Epileptor field model, and slowly propagates throughout the field. Time series at the zero location distance (where the seizure first starts) are shown in the top row for the field activity ($-u_1 + q_1$, in blue) and the slow permittivity variable (v, in red). The lower row shows a zoomed-in view of the spatiotemporal activity at seizure onset (left) and offset (right). The seizure does not end synchronously in the field, with a few spike-and-wave events propagating after seizure offset.

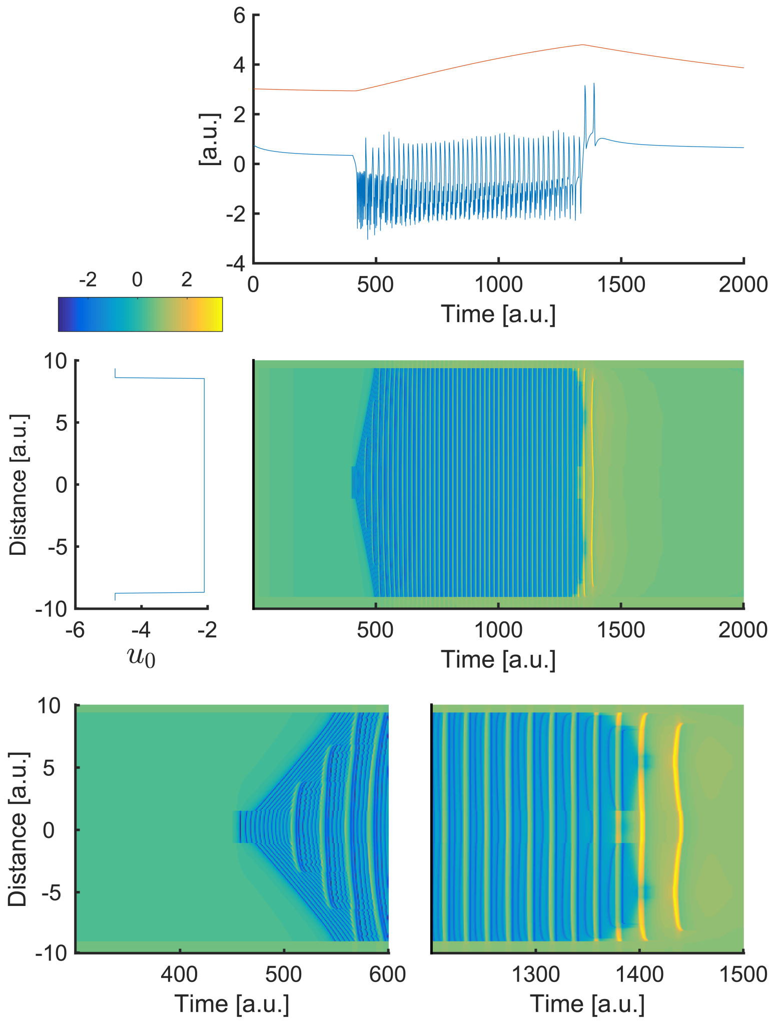

Supplementary Figure 7. The number of SWDs after seizure termination changes as a function of the strength of the coupling function γ_{12} ($\gamma_{12} = 0.5$). A single Epileptor field is simulated (middle row). Spatial values for excitability parameter and simulated spatiotemporal activity are shown on the left and right middle row respectively. The seizure starts in the central location of the Epileptor field model, and slowly propagates throughout the field. Time series at the zero location (where the seizure first starts) are shown in the top row for the field activity ($-u_1 + q_1$, in blue) and the slow permittivity variable (v, in red). The lower row shows a zoomed-in view of the spatiotemporal activity at seizure onset (left) and offset (right). Only two SWDs appear after seizure offset.

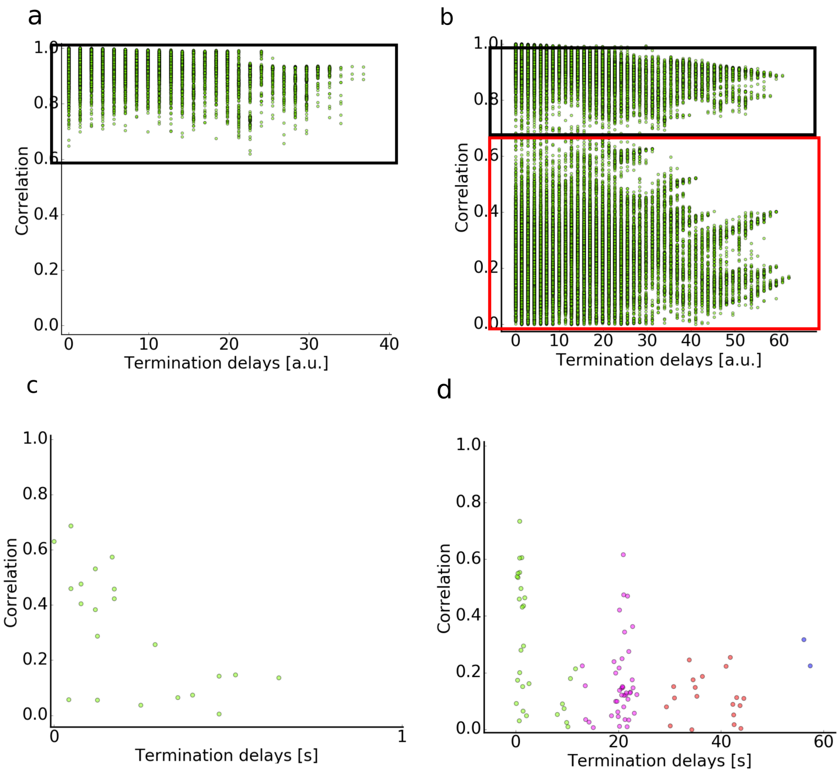

Supplementary Figure 8. Other examples of correlation values as a function of termination delays for different simulations and patients. (a) In this simulation, the seizure in the first field does not recruit the second field. All sites belong to the same field and show high correlation values. Only one cluster is found (green dots). Black rectangle: pairwise correlation for spatial locations in the same Epileptor field models. (b) In this simulation, both fields end seizure simultaneously, resulting in small and high correlation values for short termination delays. One single cluster is found by the clustering algorithm (green dots). Black (red) rectangle: pairwise correlation for spatial locations in the same (different) Epileptor field models. (c) For this recorded seizure, the seizure ends synchronously across all channels. On single cluster is found (green dots). (d) For this recorded seizure, termination delays go from 0 to 60 seconds. Four clusters are found by the clustering algorithm (green, purple, red, and blue dots).

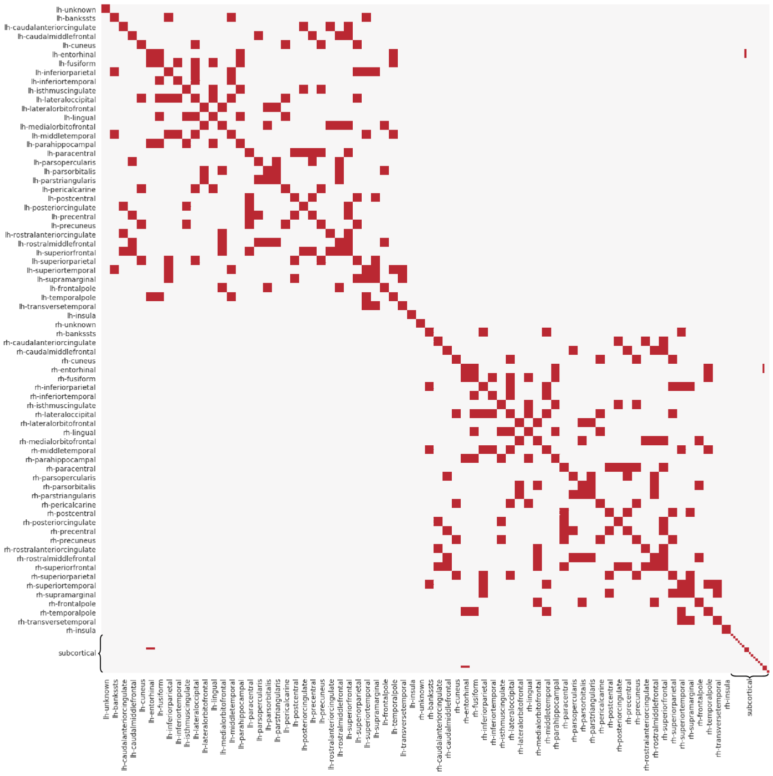

Supplementary Figure 9. Binary proximity matrix. This matrix shows if two regions are contiguous in the atlas we used (Desikan-Kiliany atlas subdivided into 297 regions, see Materials and Methods). A red pixel indicates that the corresponding row and column are contiguous in this atlas.

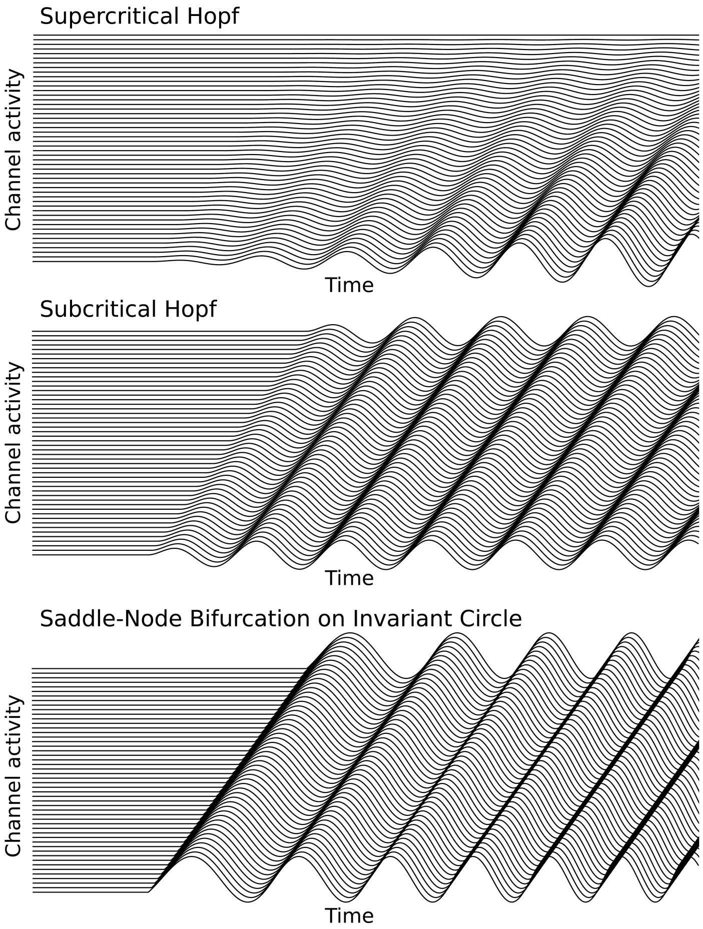

Supplementary Figure 10. Illustration of three possible scenarios of spatiotemporal propagations of focal seizures. For these three onset bifurcations (supercritical Hopf, subcritical Hopf, and saddle-node on invariant circle), oscillations appear after the onset, with each cycle propagating slowly in space. Different bifurcations come with different scaling laws of amplitude and frequency (1). For the supercritical Hopf bifurcation, amplitude grows as the square root of the distance to the bifurcation, resulting in increasing propagation extent. For the subcritical Hopf, frequencies and amplitudes are determined by the stable limit cycle. For the saddle-node bifurcation on invariant circle, frequency grows as the square root of the distance to the bifurcation. Time is along the horizontal axis. Each curve ("channel activity") refers to the activity recorded at a different location in the field.

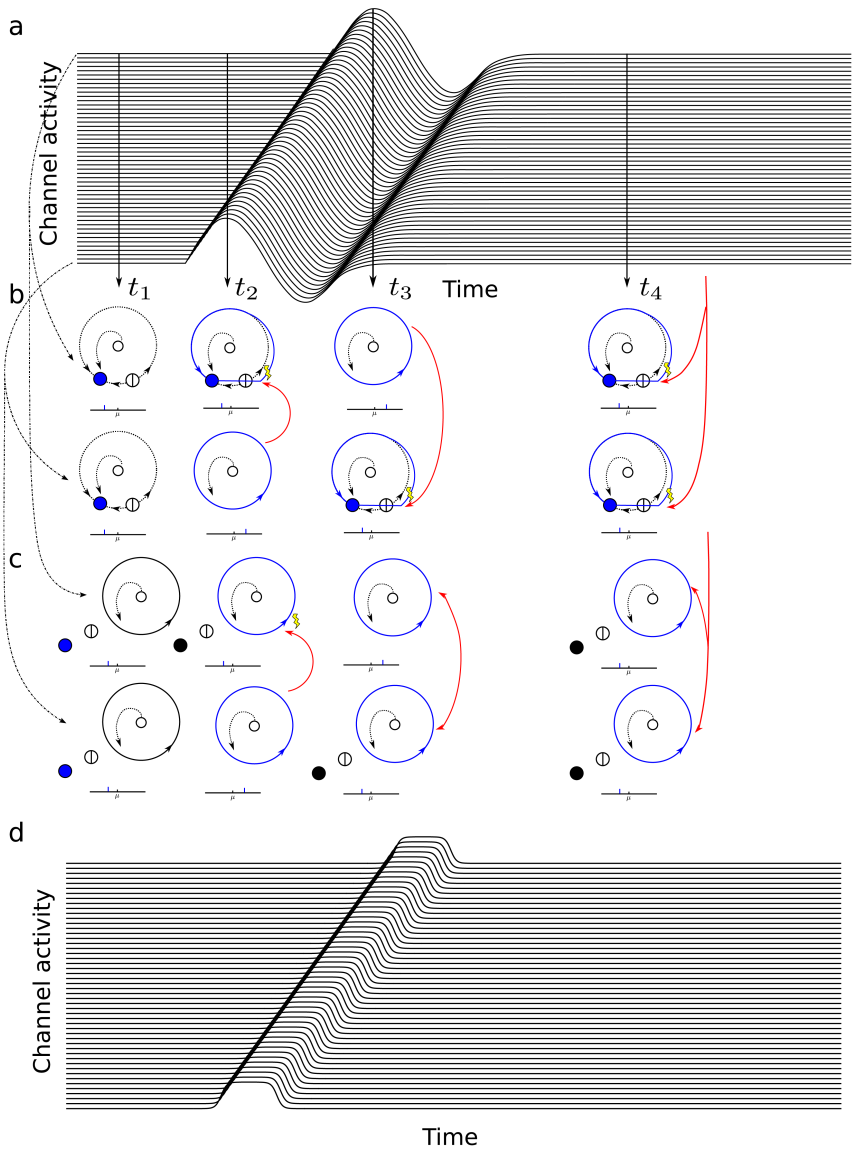

Supplementary Figure 11. Illustration of the pulse propagation scenario for ictal wavefront propagation. (a) For all three bifurcations of Supplementary Fig. 10, we can also have only one pulse propagating. Time is along the horizontal axis. Each curve refers to the activity recorded at a different location in the field. (b) Excitable media mechanism of propagation of SWDs. Phase space of the second field is shown at different time points (columns) and two different locations (rows). At time t_1, both systems are on a stable fixed point. At time t_2, the bottom system gets into an oscillatory state and send SWDs by excitation of neighboring systems (wave propagation). At time t_3, the top system enters the oscillatory state and excites the rest of the field. Finally at time t_4, both systems passively receive excitation from other parts of the field. (c) The coupled-oscillator mechanism for SWD propagation via hysteresis. Phase space of the second field is shown at different time points (columns) and two different locations (rows). At time t_1, both systems are on a stable fixed point. At time t_2, the bottom system gets into an oscillatory state and sends SWDs by excitation of neighboring systems (wave propagation). At time t_3, it is the top system that is in oscillatory state and excites the rest of the field. However, because of hysteresis, the bottom system stays in oscillatory state, resulting in coupled-oscillator dynamics. Finally at time t_4, both systems oscillate and interact with other parts of the field. (d) Other possible pulse propagation scenario with onset front immediately followed by an offset bifurcation. Time and space as in (a). Each curve ("channel activity") refers to the activity recorded at a different location in the field.

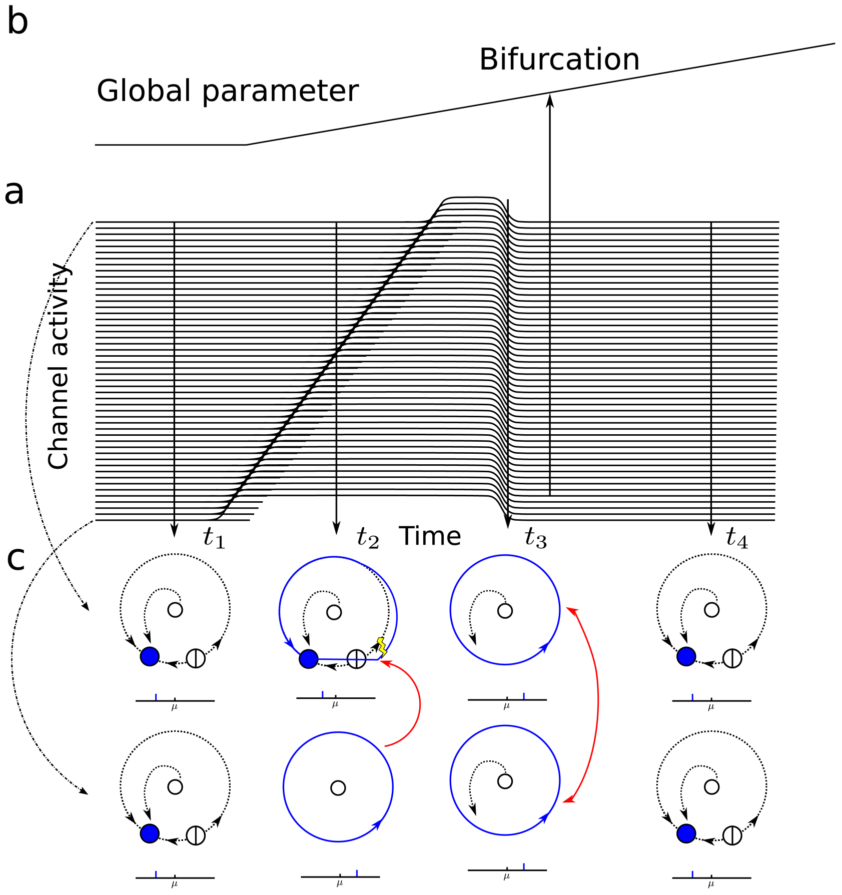

Supplementary Figure 12. Illustration of the front propagation scenario for ictal wavefront propagation. (a) A seizure propagates as a front and terminates synchronously. Each curve refers to the activity recorded at a different location in the field. (b) Possible mechanism of seizure termination: a global parameter for the field changes as the seizure evolves, and at some point crosses a bifurcation which renders the front globally unstable. (c) The coupled-oscillator mechanism as described in the Results section. Phase space of the second field is shown at different time points (columns) and two different locations (rows). At time t_1, both systems are on a stable fixed point. At time t_2, the bottom system gets into an oscillatory state and sends SWDs by excitation of neighboring systems (wave propagation). At time t_3, the top system gets into an oscillatory state as well, resulting in coupled-oscillator dynamics. Finally at time t_4, both systems return to the stable fixed point.

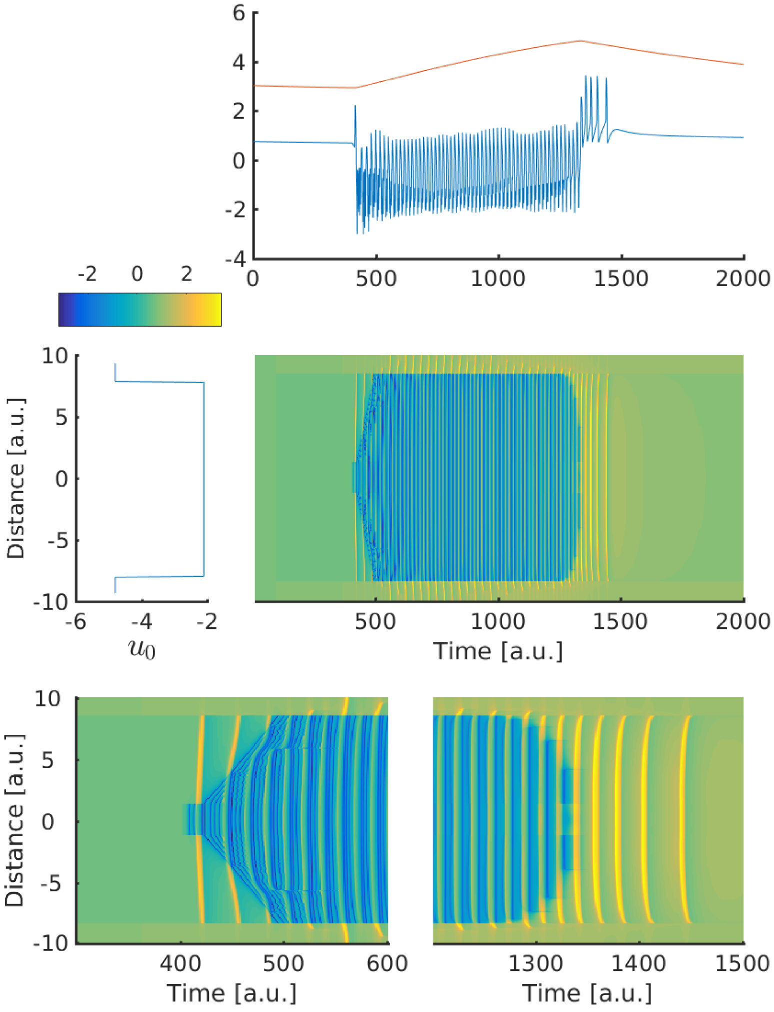

Supplementary Figure 13. Spike-and-wave discharges are shifted toward the beginning of the seizure when using the original Epileptor model value for the parameter a_{12}. In this simulation the parameter a_{12} of the temporal convolution is $a_{12} = 1$. A single Epileptor field is simulated (middle row). Spatial values for excitability parameter and simulated spatiotemporal activity are shown on the left and right middle row respectively. The seizure starts in the central location of the Epileptor field model, and slowly propagates throughout the field. Time series at the zero location (where the seizure first starts) are shown in the top row for the field activity ($-u_1 + q_1$, in blue) and the slow permittivity variable (v, in red). The lower row shows a zoomed-in view of the spatiotemporal activity at seizure onset (left) and offset (right). Preictal spikes appear before seizure onset, propagating toward easily excitable regions.

Supplementary Table 1: Patient information

Patient	Gender	Epilepsy duration (years)	Age at seizure onset (years)	Epilepsy type	Surgical procedure	Surgical outcome	MRI	Histophathology	Side	Number of seizures
AC	F	14	8	Temporal	Sr	III	Anterior temporal necrosis	Gliosis	R	4
CJ	F	14	9	Occipital	Sr	III	N	FCD type 1	L	3
CV	F	18	5	Supplementary motor area	Sr	I	N	FCD type 2	L	13
ET	F	23	7	Parietal	Sr	I	FCD SPC	FCD type 2	L	10
FB	F	16	7	Premotor	Sr	II	N	NA	R	7
FO	M	45	11	Temporo-frontal	Sr	I	FCD Fr	FCD type 2	R	1
GC	M	5	18	Temporal	Sr	III	Temporopolar hypersignal	FCD type 1	R	1
JS	M	11	18	Frontal	Sr	I	Frontal necrosis (post-trauma)	Gliosis	R	1
ML	F	10	17	Temporal	Sr	II	Hippocampal sclerosis)	NA	R	5
PC	M	15	14	Temporal	Sr	NO	N	NA	R	2
PG	M	29	7	Temporal	Sr	I	Cavernoma	Cavernoma	R	1
RB	M	28	35	Temporal	Sr	III	N	Gliosis	L	1
SF	F	24	4	Occipital	Sr	NO	PVH	NA	R	5

Th, thermocoagulation; Gk, Gamma knife; Sr, surgical resection; NO, not operated; N, normal; FCD, focal cortical dysplasia; SPC, superior parietal cortex; Fr, Frontal; PVH, periventricular nodular heterotopia; NA, not available; L, left; R, right.

Supplementary references

1. Jirsa, V. K., Stacey, W. C., Quilichini, P. P., Ivanov, A. I. & Bernard, C. On the nature of seizure dynamics. *Brain* **137**, 2210–2230 (2014).
